# *In Vitro* Coculture of Primary Human Cells to Analyze Angiogenesis, Osteogenesis, and the Inflammatory Response to Newly Developed Osteosynthesis Material for Pediatric Maxillofacial Traumatology: A Potential Pretesting Model before *In Vivo* Experiments

**DOI:** 10.1155/2023/4040504

**Published:** 2023-08-04

**Authors:** Eva Dohle, Tatjana Fecht, Tobias Wolfram, Frank Reinauer, Anke Wunder, Katja Heppe, Robert Sader, Charles James Kirkpatrick, Shahram Ghanaati

**Affiliations:** ^1^FORM, Frankfurt Orofacial Regenerative Medicine, Department for Oral, Cranio-Maxillofacial and Facial Plastic Surgery, Medical Center of the Johann Wolfgang Goethe University, Frankfurt, Germany; ^2^Karl Leibinger Medizintechnik GmbH & Co KG (KLS Martin), Tuttlingen, Germany; ^3^BioLog Heppe GmbH, Landsberg, Germany

## Abstract

During the present study, an *in vitro* coculture bone tissue mimic based on primary osteoblasts and primary endothelial cells was used for a complex and broad evaluation of a newly developed material for applications in pediatric maxillofacial traumatology. The biomaterial was composed of PDLLA (poly(D,L-lactide)) in various combinations with calcium carbonate (CC), magnesium (Mg), and chitosan (CH). Besides classical biocompatibility analyses, the present study evaluated material-dependent effects on fundamental processes that are essential for successful material integration and regeneration. Therefore, inflammation-associated factors such as E-selectin and interleukins were analyzed in the *in vitro* model system on gene expression and protein level depending on the different materials. Furthermore, in order to test the capability of vascularization of the material, the effect of the different materials on the formation of microvessel-like structures as well as the expression and release of proangiogenic factors was investigated *in vitro* in the bone coculture model. In addition, the mineralization capacity as well as the relative gene expression of osteogenic differentiation factors was analyzed in response to the different materials. As a result, the authors could assess the material combination PDLLA: CC CH as the most functionally tested material with regard to biocompatibility, inflammatory response, and microvessel-like structure formation as well as osteogenic differentiation in the *in vitro* coculture system. In conclusion, by using tissue-engineered human bone tissue equivalents as proposed here in an *in vitro* coculture model, biomaterial-mediated effects can be readily investigated. Moreover, it is proposed that these complex *in vitro* evaluations could contribute to the understanding and improvement of the development of novel materials for pediatric traumatological care for prospective clinical applications.

## 1. Introduction

The treatment of pediatric maxillofacial fractures remains a surgical challenge since fractures in children differ significantly from fractures in adults [1–3]. Those differences include general skull and face anatomy, continuously growing and developing bones, bone structure of children, tooth development, and present tooth germs as well as difficulties in cooperation of the patient during the treatment procedure [4]. The reconstruction of pediatric fractures requires in the first instance the stabilization of the fragments but, in addition, must not impair the physiological growth of the bones. However, for open reduction and internal fixation (ORIF), nonresorbable titanium osteosynthesis plates still represent the gold standard on account of their excellent biocompatibility and stability [1, 4, 5]. Nevertheless, the major disadvantage of the application of these plates is the need to be removed in a second operation very soon after the restoration, thus making a second surgical procedure necessary. The latter carries the additional risk of injury to aesthetically and functionally important structures as well as the risk of scarring, especially in the facial area. As a result, the challenge in pediatric traumatological care is the requirement for resorbable and stable osteosynthesis materials that ensure the necessary stability over a defined period of time when treating fractures in the child's facial skeleton. In addition, the material must degrade in a time frame that avoids a second surgical operation procedure [6–8]. Thus, to meet the clinical requirements, the material design should precisely correspond to the child's anatomical features by means of predetermined breaking points and fiber reinforcement. In order to meet these requirements, during this study, a newly developed osteosynthesis material engineered from PDLLA (poly(D, L-lactide)) with differing combinations with calcium carbonate (CC), magnesium (Mg), and chitosan (CH) has been developed and evaluated *in vitro*. The latter involved an endothelial/osteoblast coculture for bone tissue as the first step for the *in vitro* determination of the optimal composition of these novel materials in the field of pediatric traumatology.

Since a better understanding of bone fracture healing mechanisms can be generally assisted by *in vitro* studies, tissue-engineered human bone equivalents might significantly contribute to the understanding and improvement of developing new beneficial osteosynthesis materials in the context of pediatric maxillofacial fracture treatment [9–11]. By means of innovative *in vitro* coculture models consisting of primary osteoblasts (or fibroblasts) and primary endothelial cells, a complex and broad evaluation of newly developed materials is possible depending on the material application [12–14]. In the present basic research study, the coculture model for bone tissue consisted of human primary osteoblasts (pOB) and human primary dermal microvascular endothelial cells (HDMEC), which was then applied to fully characterize and evaluate the novel composite osteosynthesis material described previously. Besides general biocompatibility and cytotoxicity analyses, the more elaborate assessment of the materials' functionality with a focus on cellular processes associated with the implantation procedure, including inflammation, vascularization, and osteogenic differentiation, can be well achieved with this *in vitro* bone mimic model.

Various mechanisms are activated in the human organism after the implantation of a biomaterial [15–17]. The initial reaction that takes place when a biomaterial is implanted into the human body is a nonspecific inflammatory response that can range from good tolerability (biocompatibility) with a mild inflammatory reaction through a rejection reaction to complete integration of the material in the optimal case. In order to be able to make a reliable statement about the body's inflammatory reaction in response to the materials when coming into contact with the different materials, inflammation-associated factors have been analyzed at both gene and protein levels in the *in vitro* model system described in the present study. Furthermore, the rapid supply of blood vessels (vascularization) is considered to be one of the key factors in bone regeneration and is therefore crucial for a good integration of the material. Numerous published studies have shown that the cocultivation of osteoblasts and endothelial cells reliably leads to the formation of microvessel-like structures with a functional lumen [18–21]. To test the capability of vascularization of the material and to be able to make statements about the functionality of the material, the effect of the different materials on the endothelial ability to form microvessel-like structures as well as the expression and release of proangiogenic factors was investigated in the bone coculture model. In addition, with the use of this coculture model, the process of osteogenic differentiation, which is also vital for sufficient fracture healing, can also be simulated in a meaningful way. To achieve this, the mineralization capacity as well as the relative gene expression of osteogenic differentiation factors was assessed in the *in vitro* model in response to the different materials. The following study using the coculture model as a biological mimic of bone tissue will demonstrate the model's effectiveness as a novel and innovative approach to evaluate new and highly sophisticated materials for pediatric traumatological care.

## 2. Materials and Methods

### 2.1. Ethical Statement

All cells that were used for this study were obtained from excess tissue, and their application was in accordance with the principle of informed consent and approved by the responsible Ethics Commission of the state Hessen, Germany.

### 2.2. Material Composition

For the first evaluation step, the following basic materials were initially tested in terms of biocompatibility: PDLLA (poly(D, L-lactide)), PDLLA with calcium carbonate (PDLLA: CC), PDLLA with calcium carbonate and magnesium (PDLLA: CC + Mg), poly L-lactide and polyglycolic acid (PLLA: PGA) and chitosan (CH). The base polymers were used as received: PDLLA and PLLA: PGA from Evonik (Darmstadt, Germany) and biocomposite material PDLLA: CC from SchäferKalk (Hahnstätten, Germany). Magnesium alloy WE43 was used as powder formulation (MeoTech, Aachen, Germany) and combined in a speed mixer (Hauschild, Hamm, Germany) at room temperature with mixing parameters (800 to 1200 rpm) in a final weight% formulation of 90% PDLLA: CC and 10% WE43. Chitosan raw material (DD = 90%) with a molecular weight of 110 kDa was manufactured and processed by the project-affiliated partner BioLog Heppe® GmbH [22]. Therefore, chitosan was dissolved in acetic acid before starting the spinning process using a solvent wet-spinning machine (FOURNÉ, Alfter, Germany). For initial biocompatibility analyses, fibers of chitosan were manufactured in multifilaments (Yarn count: 167 tex, diameter: 23 *µ*m, and tenacity: 12.6 cN/tex). For the combination of chitosan fibers with the polymer material (PLLA: PGA, PDLLA, PDLLA: CC, and PDLLA: CC + Mg), multifilaments were manufactured with the following parameters (Yarn count: 224 tex, diameter: 32 *µ*m, and tenacity: 14.1 cN/tex). For the osteosynthesis material combination with chitosan, the corresponding basis material that forms the matrix, PDLLA: CC, and the magnesium alloy was first weighed and mixed in the speed mixer. The chitosan endless fiber was stretched in the press frame before the mixed composite material was placed on the chitosan continuous fibers. At a temperature of 80–120°C and pressure of 100–200 bar for 10–30 minutes, the composite material was pressed and a wafer was produced. The wafer was further machined in a milling device (Chiron, Tuttlingen, Germany) and was milled into coin shape structure for cell culture application in 24-well plates.

### 2.3. Primary Cells

Human primary osteoblasts (pOB) were isolated from juvenile cancellous bone fragments as already described [23]. Cells were cultivated in Dulbecco's modified Eagles' medium/nutrient mixture F-12 (Sigma-Aldrich, St. Louis, USA) supplemented with 10% FBS (Biochrom, Berlin, Germany) and 1% penicillin/streptomycin (Sigma-Aldrich, St. Louis, USA) at 37°C in a humidified atmosphere and were used up to passage 4 in this study. Human dermal microvascular endothelial cells (HDMECs) and human dermal fibroblasts (HDFs) were isolated from excess tissue of juvenile patients that underwent cleft lip reconstruction. Cells were isolated according to an established protocol [24] via several steps of enzymatic digestion and magnetic bead cell separation for endothelial cell-specific CD31. Following cell separation, HDMECs were cultivated in an endothelial growth medium (Promocell) and were used up to passage 4. Human dermal fibroblasts were obtained as the CD31-negative cell fraction and were further cultivated in Dulbecco's modified Eagle's medium (DMEM; Sigma-Aldrich, St. Louis, MO, USA). All primary cell types were characterized in a standard protocol using cell-specific immunofluorescence staining before they were used for cell experimentation. Cell culture experiments were performed with at least 3 different donors of primary cells (*n*). The individual number of samples and donors is documented in the appropriate figure legends of the results part.

### 2.4. Biocompatibility Analyses

**Figure 1:**
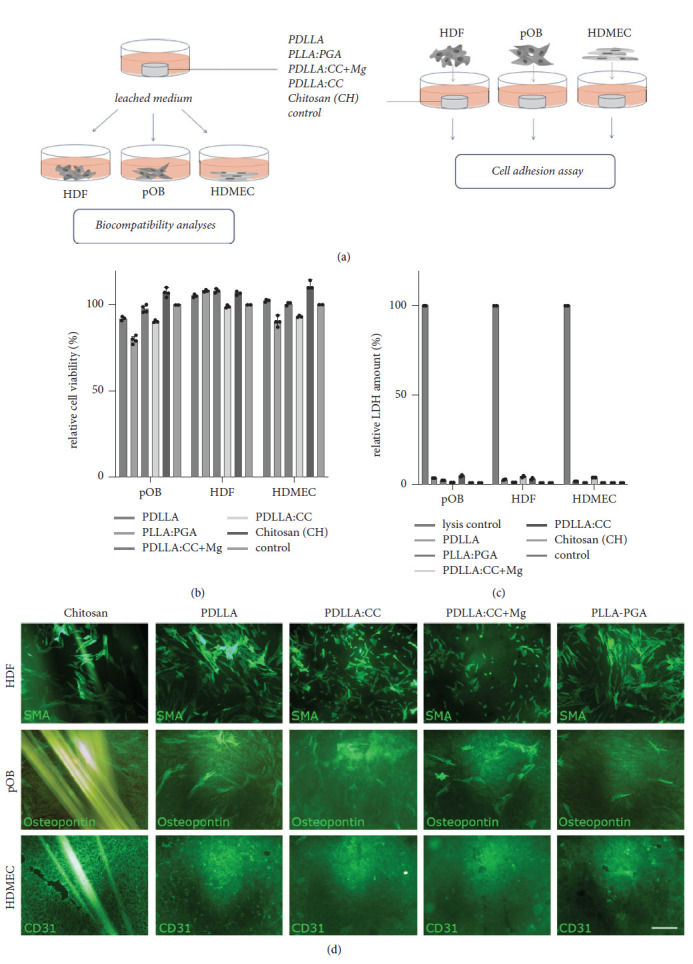
Evaluation of effects of basic material on cell viability and cell-to-material adhesion of primary cells in monoculture. (a) Graphical representation of experimental setting for initial evaluation of the materials PDLLA, PDLLA: PGA, PDLLA: CC + Mg, and Chitosan: biocompatibility analyses and cell adhesion assay were performed in monocultures of primary cells HDF, HDMEC, and pOB (*n* = 4). (b) Determination of relative cell viability compared to control using an MTS extraction assay (*n* = 4). (c) Analyzation of relative LDH amount in response to leached medium of the used materials compared to lysis control set to 100%. (d) Cell type-specific immunofluorescence staining of pOB (Osteopontin) and HDF (SMA) an HDMEC (CD31) of cells seeded on top of the appropriate materials to assess cell-to-material adhesion (*n* = 4). Scale bar in figure *D* = 150 *µ*m.

To exclude possible general cytotoxic or cell-damaging effects arising from the different basic material compositions (PLLA: PGA, PDLLA, PDLLA: CC, PDLLA: CC + Mg, and Chitosan) on the described cell types, an extraction assay on monocultures of the relevant primary cells (HDF, pOB, HDMEC) was performed prior to coculture experimentation (Figure 1(a), graphical representation). Thus, the different materials were incubated in a cell culture medium for 4 days at 37°C in an atmosphere of 5% CO_2_ and 95% oxygen. Primary fibroblasts, primary osteoblasts, and HDMEC were seeded on 96-well plates (5.000 cells/well). 200 *µ*l of the leached medium (extraction medium) was added to the preseeded cells, and cells were incubated for an additional four days before analyzing for cell viability using MTS CellTiter 96®AQ_ueous_ One Solution Cell Proliferation Assay (Promega, Madison, USA) according to the manufacturer's protocol. Absorbance was measured at 490 nm in a microplate reader (GENios plus, TECAN, Crailsheim, Germany). Furthermore, an LDH assay was performed according to the manufacturer's protocol (Sigma-Aldrich, St. Louis, MO, USA) to determine the amount of dead cells after incubation of the cells with the leached medium of the different basic materials.

### 2.5. Adhesion Assay

For initial material evaluation, the adhesion capacity of the appropriate cells in monoculture (HDMECs, pOB, and HDF) on the different basic materials was analyzed (Figure 1(a), graphical representation). In achieving this, PLLA: PGA, PDLLA, PDLLA: CC, and PDLLA: CC + Mg as well as solely chitosan fibers were placed in 24-well plates before the primary cells were seeded on top of the materials. Cell/material complexes were cultivated for 7 days before cells were fixed using 3.7% PFA and further processed for cell-type-specific immunofluorescence staining and microscopic evaluation.

### 2.6. Coculture Experimentation

**Figure 2:**
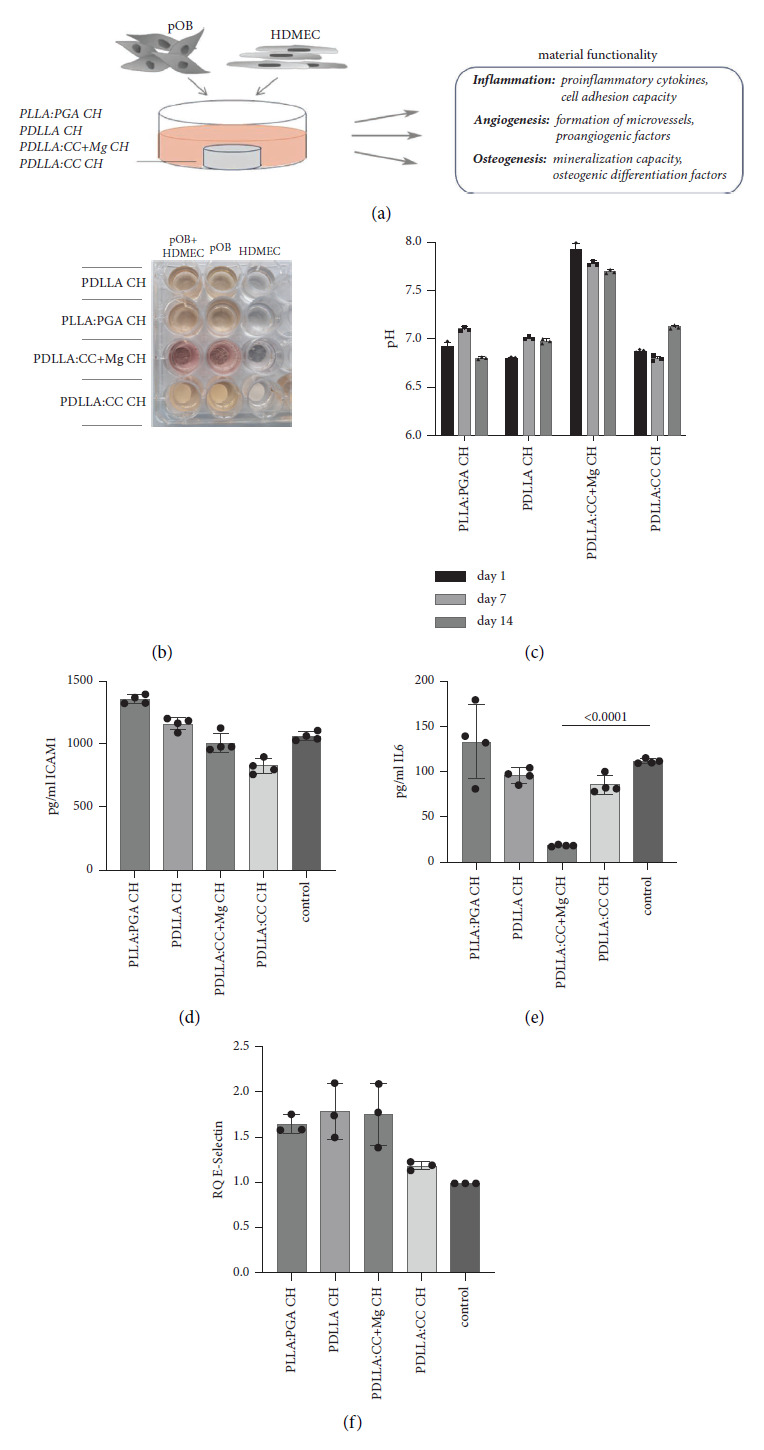
Evaluation of inflammation-associated factors in an *in vitro* coculture model for bone tissue seeded on differently composed materials in combination with chitosan. (a) Schematic overview of experimental settings for *in vitro* coculture experimentation for functional evaluation (inflammation, angiogenesis, and osteogenesis) of base material combined with chitosan (PDLLA: PGA CH, PDLLA CH, PDLLA: CC CH, and PDLLA: CC + Mg CH) (*n* = 4). (b) Overview of the material-coculture and monoculture combination in 24-well plates. (c) Determination of pH in cocultures seeded on the appropriate materials after 1, 7, and 14 days of cultivation (*n* = 3). (d) Evaluation of ICAM-1 in cell culture supernatants after 14 days of cultivation. (e) Evaluation of IL-6 in supernatants after 14 days of cultivation (*n* = 4). (f) Relative quantification of gene expression of E-selectin in cocultures seeded on different materials after 14 days of cultivation (*n* = 3).

For the *in vitro* coculture model for bone tissue, HDMEC and pOB were mixed in a 1 : 2 ratio and then seeded on top of the materials. Coculture experimentation was performed to evaluate the materials' functionality of the newly manufactured materials combined with chitosan (CH): PLLA: PGA CH, PDLLA CH, PDLLA: CC + Mg CH, and PDLLA: CC CH (Figure 2(a), graphical representation). The different materials were placed in 24-well plates before 20.000 pOB and 20.000 HDMEC were mixed and added to the materials without an additional coating of the materials and further cocultivated for 14 days. Due to the higher growth sensitivity of endothelial cells in the coculture system, cells were cultivated in an endothelial cell growth medium with medium change twice per week. After 14 days of cultivation, supernatants of coculture/material combinations were collected for further experimentation (ELISA) and stored at −80°C until analyzation. In addition, cells were lysed and processed for mRNA isolation and quantitative real-time PCR as well as fixed for immunofluorescence staining (CD31) and alizarin red staining. During the course of cultivation, pH values were measured and documented in the supernatants of the different material-cell complexes using a pH measurement device (FiveEasy™FE20, Mettler-Toledo AG, Schwerzenbach, Switzerland) on day 1, day 7, and day 14 of cultivation.

### 2.7. Immunofluorescence Staining

For immunofluorescence staining, cells were fixed in 4% buffered formalin (Roti-Histofix 4% acid-free pH7, Carl-Roth, Germany), permeabilized with 0.5% Triton X/PBS, and washed three times with PBS before incubation with the following antibodies: mouse anti-human CD31 (1 : 40, Dako), mouse anti-human osteopontin (1 : 100; Dako), and mouse anti-human alpha-smooth muscle actin (1 : 100, Dako) diluted in 1% bovine serum albumin/PBS solution according to previously published studies [12, 19]. The stained cell cultures were examined using a fluorescence microscope (Nikon Eclipse TS100; Nikon, Düsseldorf, Germany). In addition, immunofluorescence staining for CD31, documenting the formation of microvessel-like structures by endothelial cells in the coculture, was quantitively analyzed using the image processing software NIS Elements (Nikon, Düsseldorf, Germany) as already described elsewhere [12].

### 2.8. Osteogenesis Assay

To quantify the mineralization capacity of the cocultures seeded with the different materials, an osteogenesis quantification kit was used according to the manufacturer's protocol (Merck Millipore, Darmstadt, Germany). Therefore, after removing the material, cocultures at the bottom of the cell culture plastic were incubated with an alizarin red staining solution for at least 20 minutes before the excess dye was removed. Washed and stained cells were then assessed and documented using a light microscope. After visual inspection and documentation of the stained cells, alizarin red was removed from the cells via acetic acid (10%) before the stain was quantified using a microplate reader (OD_405_). Alizarin concentration was defined as *µ*M.

### 2.9. Growth Factor and Cytokine Determination

Cell culture supernatants of the coculture/material complexes were collected after 14 days of cultivation and subsequently analyzed for growth factor and cytokine concentration using ELISA Development Systems according to the manufacturer's protocol (R&D Systems). For visualization of the protein content in the supernatants, a streptavidin-HRP colorimetric reaction was used, and the optical density was measured using a microplate reader (Tecan, Crailsheim, Germany) at a wavelength of 450 nm. The following ELISA DuoSets were used in this study: vascular endothelial growth factor (VEGF), interleukin 6 (IL-6), intercellular adhesion molecule 1 (ICAM-1), osteoprotegerin (OPG), and interleukin 8 (IL-8).

### 2.10. Gene Expression Analysis

RNA isolation was performed using RNeasy micro kit according to the manufacturer's instructions (Qiagen). 1 *µ*g of extracted RNA was used to transcribe into complementary DNA (cDNA) according to a standard protocol using Omniscript Reverse Transcription Kit (Qiagen). For quantitative real-time PCR, the following primers were used during this study: E-selectin, osteonectin, and alkaline phosphatase (ALP). For quantitative real-time PCR (qRT PCR), 4 ng cDNA was used for one reaction and with the following cycler program: 95°C 10 min, 95°C 15 sec, 60°C 1 min, 40 m cycles. To specify the length of the DNA fragments, a dissociation stage was added to the programme. qRT PCR was performed in triplicate, and relative gene expression was determined using the delta-delta CT method. Gene expression was compared by setting control cultures to 1 (reference value) as indicated in the relevant figures.

### 2.11. Statistical Analyses

All experiments were performed with at least three different donors of primary cells (*n*) as indicated in the appropriate figure legends, and individual data points for each “*n*” are depicted in the diagrams. Results were calculated as mean ± standard deviation (SD) and were evaluated for significant differences with one-way ANOVA and post-hoc testing (Dunnett's multiple comparison test) using GraphPad Prism 9.0 software (GraphPad Software Inc.). Statistically significant differences (*p* values) were directly documented in the diagrams of the figures.

## 3. Results

### 3.1. Evaluation of Material Effects on Cell Viability and Cell-to-Material Adhesion of Primary Cells in Monoculture: HDF, HDMEC, and pOB

To exclude possible cytotoxic effects arising from the differently composed basic materials, the relevant primary cell types such as human dermal fibroblasts (HDFs), human primary osteoblasts (pOBs), and human dermal microvascular endothelial cells (HDMECs) were first used for analyzation in monocultures, respectively. Therefore, the sterilized samples of the following basic materials (combination) PDLLA, PLLA: PGA, PDLLA: CC + Mg, PDLLA: CC, and chitosan were initially examined with respect to possible cytotoxic effects in monocultures of the appropriate cell types (Figure 1(a)). For this purpose, HDMEC, pOB, and HDF were cultivated separately in an extract of the different materials before the viability of the cells was determined using MTS assay and LDH assay (Figures 1(b) and 1(c)). No negative effects of the extraction media (leached medium) on the cells in monoculture could be determined when cells were cultivated in the different extraction media for 4 days. The direct contact of the cells with the materials followed by cell-type-specific immunofluorescence staining for the appropriate cell types (CD31, osteopontin, SMA) also confirmed consistently good cell-to-material adhesion and a regular cell-type-specific morphology of all cell types seeded on the materials (Figure 1(d)). Physiological pH values ranging from pH 6.8 to pH 7.1 could be determined when cocultures were cultivated for 1, 7, and 14 days on all tested materials except for the PDLLA group when combined with the Mg alloy (Figure 2(c)). Although the material composition with PDLLA: CC + Mg CH (PDLLA: CC + Mg CH) led to the general increase in pH from ∼pH 7 to ∼pH 7.8 as demonstrated by discoloration of the pH indicator phenol red supplemented to the cell culture medium (Figure 2(b)) and finally documented using a pH measurement device (Figure 2(c)) after 1, 7, and 14 days of cultivation, all analyzed materials displayed good biocompatibility and met the basic requirements of an osteosynthesis material. The lack of cytotoxicity permitted further analyses with regard to the functionality of the materials.

### 3.2. Evaluation of Inflammation-Associated Factors in the *In Vitro* Coculture Model for Bone Tissue Seeded on Differently Composed Materials in Combination with Chitosan

For evaluation of material functionality, the different polymers PLLA: PGA, PDLLA, PDLLA: CC, and PDLLA: CC + Mg in combination with chitosan were characterized using a primary cell coculture model for bone tissue *in vitro*. For the functional evaluation, the coculture system consisting of primary osteoblasts (pOBs) and primary endothelial cells (HDMECs) was used. For the cell-biological characterization, the established coculture was incubated on the respective samples for 14 days before various tests were carried out with regard to three main functional parameters, namely, the processes of inflammation, angiogenesis, and osteogenic differentiation (graphical scheme in Figure 2(a)).

To investigate a possible material-mediated induction of an inflammatory response of the coculture, the cell culture supernatants were analyzed for the proinflammatory factors ICAM-1 and IL-6 (Figures 2(d) and 2(e)). Although no significant difference could be observed in the appropriate cell culture supernatants, ICAM-1 was found to be less concentrated in PDLLA: CC + Mg CH and PDLLA: CC CH group supernatants compared to controls. A slight increase in ICAM-1 release could be assessed in supernatants of PLLA: PGA CH and PDLLA CH when combined with the coculture. Interleukin-6, another proinflammatory cytokine, was significantly lower in supernatants of coculture material complexes of PDLLA: CC + Mg CH (Figure 2(e)) compared to all other analyzed supernatants. In addition, relative gene expression of E-selectin was assessed in the appropriate cultivated cell-material complexes after 14 days of cultivation (Figure 2(f)) and revealed no significant differences in relative quantification of the expression of E-selectin among the tested experimental groups.

### 3.3. Analysis of Microvessel-Like Structure Formation and Proangiogenic Growth Factor Production in the *In Vitro* Coculture Model for Bone Tissue Seeded on Differently Composed Materials in Combination with Chitosan

**Figure 3:**
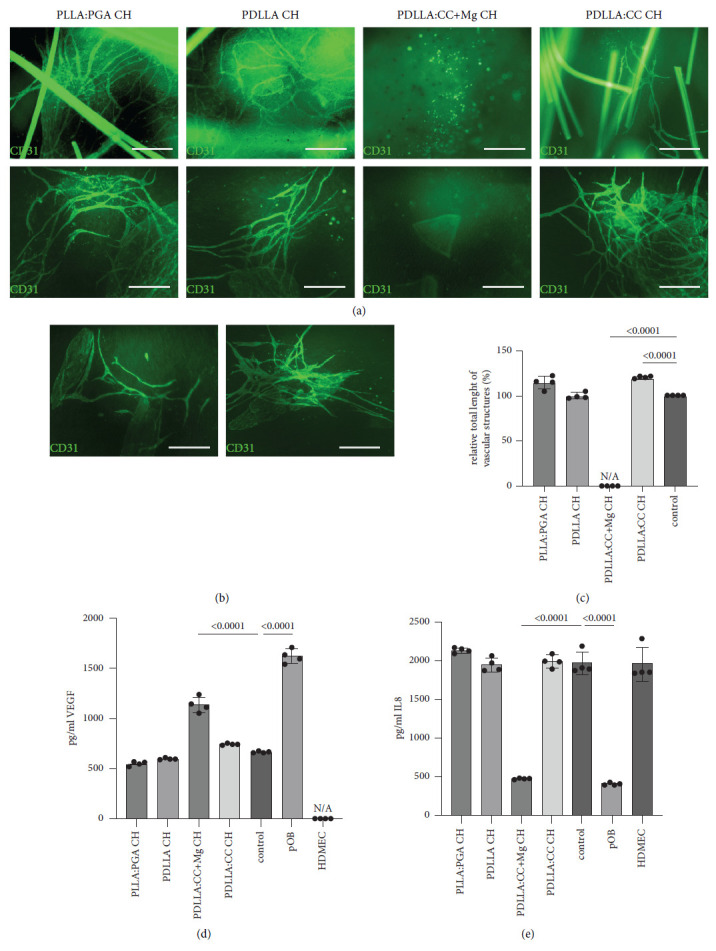
Analysis of microvessel-like structure formation and proangiogenic growth factor production in an *in vitro* coculture model for bone tissue seeded on differently composed materials in combination with chitosan. (a) Endothelial cell type-specific immunofluorescence staining for CD31 of cocultures seeded on top of the material (upper row) and cells at the bottom of the cell culture plate (lower row) after 14 days of cocultivation (*n* = 4). (b) Control cocultures seeded on cell culture plastic without material (*n* = 4). (c) Quantification of the formation of microvessel-like structures (*n* = 4). (d) Evaluation of VEGF concentration in cell culture supernatants of cocultures seeded on the different materials compared to control cocultures as well as to the appropriate cells in monoculture (*n* = 4). (e) Evaluation of IL-8 concentration in cell culture supernatants of cocultures seeded on the different materials compared to control cocultures as well as to the appropriate cells in monoculture (*n* = 4). Scale bars = 150 *µ*m.

To evaluate the capability of the endothelial cells to form microvessel-like structures in the coculture system seeded on the different polymers combined with chitosan (PLLA: PGA CH, PDLLA CH, PDLLA: CC + Mg CH, PDLLA: CC CH), cocultures were cultivated for 14 days before cells were stained immunofluorescently for the endothelial marker CD31 to document the formation of microvessel-like structures in the coculture compared to control (Figures 3(a) and 3(b)). Although the immunostain of the coculture directly on the materials showed background exposure due to autofluorescence of the materials, angiogenic structures were clearly defined when cells were combined with PLLA: PGA CH and PDLLA CH as well as with PDLLA: CC CH (Figure 3(a), upper row). In contrast, no microvessel-like structure formation could be documented when cocultures were seeded on top of PDLLA: CC + Mg CH material (Figure 3(a), upper row). In this group, endothelial cells failed to form angiogenic structures and did not even exhibit the normal endothelial cell type-specific morphology. In addition, the bottom of the cell culture plates of all experimental groups was also examined for possible CD31-positive cells that might have migrated from the material to the well plate. The results confirmed those from the direct evaluation (Figure 3(a), lower row). The induction of microvessel-like structure formation could be observed in cocultures of all analyzed experimental groups except for cocultures in combination with PDLLA: CC + Mg CH (Figure 3(a), lower row). The total length of microvessel-like structures was comparatively quantified using an image analysis program and confirmed the visual results from the immunostaining. The combination of the coculture with PDLLA: CC CH revealed the highest value (total length in pixels) of microvessel-like structures compared to the other analyzed material-cell groups (Figure 3(c)). No formation of angiogenic structures could be quantified in the PDLLA: CC + Mg CH group. The determination of the proangiogenic factors VEGF and IL-8 in the cell culture supernatants of the different coculture/material complexes revealed significant differences among cocultures seeded on the different scaffold and control cocultures (Figures 3(d) and 3(e)). Significantly higher release of VEGF into cell culture supernatants could be measured in the PDLLA: CC + Mg CH experimental group compared to all other groups, except for the VEGF release of the control pOB supernatants alone (Figure 3(d)). In contrast to the PDLLA: CC + Mg CH group, VEGF release of the cells seeded on the other tested materials ranged at a similar lower level. Analysis of the release of the proangiogenic cytokine interleukin-8 revealed the significantly lowest concentration in the PDLLA: CC + Mg CH group combined with the coculture and compared to all other analyzed experimental groups (Figure 3(e)).

### 3.4. Effect of Different Material Combinations with Chitosan on Osteogenic Differentiation Capacity in the *In Vitro* Coculture Model for Bone Tissue: Mineralization and Osteogenic Differentiation Factor Analyses

**Figure 4:**
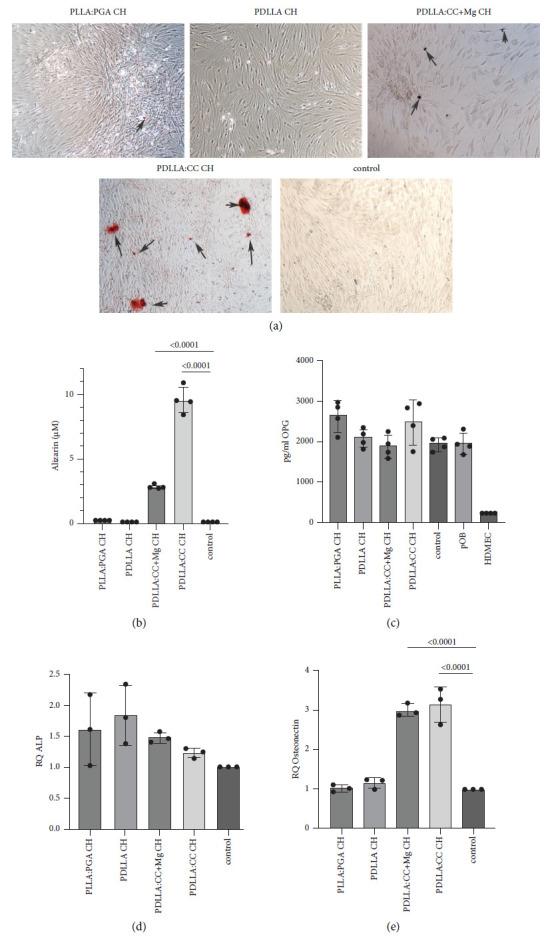
Effect of different material combinations with chitosan on osteogenic differentiation capacity in an *in vitro* coculture model for bone tissue: mineralization and osteogenic differentiation factor analyses. (a) Alizarin red staining of cocultures combined with the different materials and cultivated for 14 days (*n* = 4). (b) Quantification of the alizarin red staining (*n* = 4). (c) Evaluation of osteoprotegerin (OPG) concentration in cell culture supernatants of cocultures seeded on the different materials compared to control cocultures as well as to the appropriate cells in monoculture (*n* = 4). (d) Relative quantification of gene expression of alkaline phosphatase (ALP) in cocultures seeded on different materials after 14 days of cultivation (*n* = 3). (e) Relative quantification of gene expression of osteonectin in cocultures seeded on different materials after 14 days of cultivation (*n* = 3).

The determination of the mineralization of the osteoblasts as a marker for osteogenic differentiation in the coculture dependent on the differently composed materials PLLA: PGA CH, PDLLA: CH, PDLLA: CC + Mg CH was investigated using alizarin red staining of the cells grown on cell culture plastic with indirect contact to the material. A clear and significant initial mineralization of the osteoblasts in the coculture could be observed when cells were cultivated on PDLLA: CC CH and PDLLA: CC + Mg CH as documented by bright red areas showing the calcified primary osteoblasts (arrows Figure 4(a)). No or less alizarin red-stained-positive calcified cells could be detected in the control and when cells were combined with PLLA: PGA CH and PDLLA CH. The visual results could also be confirmed by quantification of the alizarin stain (Figure 4(b)). Analysing the supernatants of the coculture/material complexes for release of osteoprotegerin after 14 days of cocultivation revealed similar concentrations in all analyzed cell/material groups, except for the control endothelial cells in monoculture (Figure 4(c)). Relative quantification of gene expression profile documented a significant upregulation of the late osteogenic differentiation marker osteonectin when cocultures were cultivated on PDLLA: CC CH and PDLLA: CC + Mg CH compared to the cultivation of the cells on the other materials and compared to the control (Figure 4(e)). Although the results were not significant, alkaline phosphatase (ALP) gene expression, an early marker for osteogenic differentiation was found to be slightly upregulated in all analyzed coculture/material complexes compared to the control without material (Figure 4(d)).

## 4. Discussion

In the present basic research study, an *in vitro* coculture model for bone tissue was used to fully characterize and evaluate a newly developed osteosynthesis material engineered from PDLLA (poly(D,L-lactide)) together with calcium carbonate (CC), magnesium (Mg), and chitosan (CH) adapted to the specific requirements of pediatric maxillofacial traumatology. However, to date, only a few osteosynthesis plates are available specifically for use in pediatric care, and the development of osteosynthesis plates that are stable but can still be degraded quickly at defined points would be an important and forward-looking step in the pediatric treatment of facial fractures. Currently, there are few resorbable osteosynthesis plates available for clinical use. These material classes are made from polylactides (PLA), polyglycolides, and their copolymers [9, 25]. Due to their resorbability, these materials circumvent the disadvantages of titanium plates, especially the problem of growth inhibition and elimination of the need for another operation to remove the material.

Before a novel biomaterial can be used as a medical device, *in vitro* proof of its biocompatibility in mandatory cell culture tests according to defined international standard protocols is the first essential step. However, currently proposed *in vitro* methods for this evaluation of biocompatibility fail to represent the physiological situation, since genetically modified single-cell lines are generally used for the experiments and do not mirror the physiological situation in healthy tissue [26, 27]. During the present study, initial classical biocompatibility analyses that were performed with primary monocultures of primary osteoblasts, primary fibroblasts, and primary endothelial cells to make a first selection were able to exclude cytotoxic effects of the pure basic materials composed of the polymer combined with calcium carbonate and/or magnesium alloy on the appropriate primary cells in monoculture. Cell-to-material adhesion of each cell type seeded separately on the basic materials confirmed the results of cytotoxicity assays by exhibiting overall good cell adhesion of the tested cell monocultures on all used materials. Although a single cell type can give a first indication of the possible cytotoxicity of a tested material or one of its components, the prognostic statement on how the material will affect human tissue after implantation is very limited [28]. The integration of a biomaterial such as the here proposed osteosynthesis materials for application in pediatric maxillofacial traumatology strongly depends on the complex cellular interaction of different cell types that come into contact with the material after implantation. Currently, those cellular dynamics and cell interactions are still poorly represented when assessing a biomaterial *in vitro*. Nevertheless, to evaluate the material's functionality after implantation, *in vitro* tests require a cellular microenvironment that consists of more than one cell type and thus is more comparable to the physiological bone tissue. In this context, establishing and using an *in vitro* coculture model consisting of primary osteoblasts and primary endothelial cells can be strongly recommended to mimic the *in vivo* situation and might serve as a reliable bone replicate *in vitro*. Using this coculture model, the major findings of the study were as follows: (1) none of the tested materials leads to a significantly increased release or upregulated gene expression of inflammation-associated factors; (2) induction of vessel-like structures could be detected in all tested material/coculture groups except for the PDLLA: CC + Mg CH–coculture combination; and (3) significantly increased osteogenic differentiation could be assessed solely in PDLLA: CC + Mg CH–cocultures as well as in PDLLA: CC CH–cocultures.

In general, a coculture is a very dynamic system, which is controlled by various growth factors and by the way cells communicate with each other similar to the *in vivo* situation, a process known as cellular crosstalk [21]. During the last decade, several coculture systems related to bone regeneration have been developed [29–32]. Especially, endothelial/osteoblast coculture models are able to simulate the physiological situation around the implant site, as both endothelial and osteoblastic cells are the key regulators for bone regeneration processes after trauma. In addition, endothelial/osteoblast coculture models represent a convenient way to study cell-biomaterial interactions with regard to the possible induction of inflammatory processes. The implantation of a biomaterial always involves tissue trauma which results in a physiological inflammatory response, coupled with a wound healing and tissue regeneration reaction [33]. Although any cytotoxic effect of the basic materials on the used cell types in monoculture could be excluded using biocompatibility and cellular adhesion assays, additional proinflammatory factors and cytokines were analyzed at gene and protein expression level in additional experiments to exclude possible prolonged tissue injury via chronic inflammation after the implantation process. The proinflammatory factors ICAM-1, interleukin-6 (Il-6), and E-selectin were consistently on a similar same level in all tested coculture/material groups or were even lower compared to control cocultures without material contact. The release of proinflammatory factors such as endothelial-cell-based E-selectin is an excellent monitor of the body's early reaction to a biomaterial [34] and can thus be used to assess biomaterial components with respect to unwanted biological reactions. This gives valuable input for the development or improvement of the material.

Angiogenesis, the formation of new blood microvessels, is required for the regeneration process in order to transport nutrients and oxygen to the implant site and is therefore initiated immediately after tissue injury [35]. Microvascular endothelial cells are the basic component of blood vessels, and their function with regard to bone regeneration is essential for the formation of new vessels [36]. When angiogenesis fails, peri-implant tissue will undergo ischemic changes. In the present study, the angiogenic capability of endothelial cells in the coculture consisting of primary HDMEC and primary osteoblasts revealed a clear induction of microvessel-like structure formation after 14 days of cocultivation. This was observed in the following tested material compositions: PDLLA CH, PDLLA: CC CH, and PLLA: PGA CH. In contrast, a lack of formation of microvessel-like structures was found when cocultures were cultivated on PDLLA: CC + Mg CH, only differing in the content of magnesium (Mg) that has been integrated within the material. The included magnesium appears to lead to generally lower cell numbers of endothelial cells in the coculture, accompanied by a higher cell number of osteoblasts in this group compared to the other materials. This effect could be confirmed by a significantly higher release of pOB-derived VEGF in the coculture cultivated on PDLLA: CC + Mg CH scaffolds as well as a significantly lower amount of endothelial cell type-specific interleukin-8 release into the coculture supernatants. Optimal integration of an osteosynthesis material strongly depends on a good balance of cell proliferation and differentiation of the different cell types in the peri-implant tissue. Therefore, the material composition must be chosen in such a way that neither of the two cell types has an advantage or disadvantage in cell growth, proliferation, and differentiation. The magnesium alloy that was included in the PDLLA: CC CH scaffolds did not show any cytotoxic effect on the endothelial cells in monoculture but seemed to promote a significant selective advantage for the primary osteoblasts within the coculture systems. Thus, the cell ratio was skewed in favour of the osteoblasts, leading to a complete overgrowth by the pOB when cocultivated with HDMEC on PDLLA: CC + Mg CH scaffolds. It is already known that magnesium-based biomaterials might increase the process of osteogenesis, promotion of osteoblast adhesion and motility, and positive immunomodulation, as well as angiogenesis [37]. The positive effect on osteogenic differentiation of pOB in the coculture could be observed in the scaffolds combined with the Mg alloy documented by positive alizarin red staining and quantification. Nevertheless, the highest induction of mineralization was found when cocultures were seeded on PDLLA: CC CH scaffolds. Calcium carbonate is known to promote bone regeneration, as it can be converted into hydroxyapatite on coming into contact with phosphate solutions, thus serving as a source of apatite important for bone repair [38, 39]. The osteoconductive qualities of calcium carbonate are well known, and it has been suggested that it can trigger stem cells or progenitors to differentiate into osteoblasts [40, 41]. Although early osteogenic differentiation markers were not significantly changed at protein and gene expression levels in the different coculture/material complexes, the relative gene expression of the late osteogenic marker, osteonectin, was found to be significantly upregulated in the calcium carbonate material groups, documenting an ongoing osteogenic differentiation process induced by those materials in the coculture.

## 5. Conclusion

By using the described coculture system consisting of primary osteoblasts and dermal microvascular endothelial cells, the authors were able to evaluate various material combinations of newly developed osteosynthesis materials for pediatric maxillofacial traumatology and could assess PDLLA: CC CH as the most functionally tested material. The experimental study focused on biocompatibility, inflammation, blood vessel formation, and osteogenic differentiation. Complex *in vitro* models, like the proposed coculture bone mimic, allow a first complete evaluation of the manufactured material, as the essential aspects occurring after implantation, especially inflammation, angiogenesis, and osteogenic differentiation, can be assessed in a more *in vivo*-like model than is possible with monocultures. This in turn deepens our understanding of biological and cellular reactions at the tissue-biomaterial interface and provides valuable input to help modulate and adapt the material to achieve the desired biological functions. Such models which permit cellular crosstalk are necessary for the further development and optimization of novel osteosynthesis materials for the special requirements in pediatric maxillofacial traumatology.

## Data Availability

All generated or analyzed data during the study are included in the manuscript.
